# Ketogenic diet for super-refractory status epilepticus (SRSE) with NORSE and FIRES: Single tertiary center experience and literature data

**DOI:** 10.3389/fneur.2023.1134827

**Published:** 2023-04-13

**Authors:** Rima Nabbout, Sara Matricardi, Paola De Liso, Olivier Dulac, Mehdi Oualha

**Affiliations:** ^1^Reference Center for Rare Epilepsies, Department of Pediatric Neurology, Necker-Enfants Malades Hospital, Assistance Publique Hôpitaux de Paris, University Paris Cité, Member of ERN EpiCARE, Paris, France; ^2^Imagine Institute, National Institute of Health and Medical Research, Mixed Unit of Research 1163, University Paris Cité, Paris, France; ^3^Department of Pediatrics, University of Chieti, Chieti, Italy; ^4^Neurology Unit, Department of Neuroscience, Bambino Gesù Children's Hospital, Member of ERN EpiCARE, Rome, Italy; ^5^Pediatric Intensive Care Unit, Necker-Enfants Malades Hospital, Assistance Publique Hôpitaux de Paris, Université de Paris, Paris, France

**Keywords:** NORSE, New-Onset Refractory Status Epilepticus, FIRES, febrile infection-related epilepsy syndrome, SRSE, super refractory status epilepticus, ketogenic diet, KD

## Abstract

**Background and purpose:**

Ketogenic diet (KD) is an emerging treatment option for super-refractory status epilepticus (SRSE). We evaluated the effectiveness of KD in patients presenting SRSE including NORSE (and its subcategory FIRES).

**Methods:**

A retrospective review of the medical records was performed at the Necker Enfants Malades Hospital. All children with SRSE in whom KD was started during the last 10 years were included. A systematic search was carried out for all study designs, including at least one patient of any age with SRSE in whom KD was started. The primary outcome was the responder rate and Kaplan–Meier survival curves were generated for the time-to-KD response. As secondary outcomes, Cox proportional hazard models were created to assess the impact of NORSE-related factors on KD efficacy.

**Results:**

Sixteen children received KD for treatment of SRSE, and three had NORSE presentation (one infectious etiology, two FIRES). In medical literature, 1,613 records were initially identified, and 75 were selected for review. We selected 276 patients receiving KD during SRSE. The most common etiology of SRSE was acute symptomatic (21.3%), among these patients, 67.7% presented with NORSE of immune and infectious etiologies. Other etiologies were remote symptomatic (6.8%), progressive symptomatic (6.1%), and SE in defined electroclinical syndromes (14.8%), including two patients with genetic etiology and NORSE presentation. The etiology was unknown in 50.7% of the patients presenting with cryptogenic NORSE, of which 102 presented with FIRES. Overall, most patients with NORSE benefit from KD (*p* < 0.004), but they needed a longer time to achieve RSE resolution after starting KD compared with other non-NORSE SRSE (*p* = 0.001). The response to KD in the NORSE group with identified etiology compared to the cryptogenic NORSE was significantly higher (*p* = 0.01), and the time to achieve SE resolution after starting KD was shorter (*p* = 0.04).

**Conclusions:**

The search for underlying etiology should help to a better-targeted therapy. KD can have good efficacy in NORSE; however, the time to achieve SE resolution seems to be longer in cryptogenic cases. These findings highlight the therapeutic role of KD in NORSE, even though this favorable response needs to be better confirmed in prospective controlled studies.

## 1. Introduction

Status epilepticus (SE) is a potentially life-threatening condition resulting either from the failure of the natural homeostatic suppressing mechanisms responsible for seizure termination or from the initiation of mechanisms leading to abnormally prolonged seizure activity (1). About 31%−43% of the patients with SE are not controlled with first- and second-line treatments and enter in refractory SE (RSE), requiring intravenous anesthetic drugs (2). About 15% of the patients will progress further to super-refractory SE (SRSE), defined as SE that persists for more than 24 h after the initiation of anesthesia or recurs on the reduction or withdrawal of anesthetic drugs (3).

New-Onset Refractory Status Epilepticus (NORSE) is the clinical presentation describing a patient without active epilepsy or other preexisting relevant neurological disorder occurring without age limitation. It is characterized by *de novo* onset of RSE without a clear acute or active structural, toxic, or metabolic cause (4). The diagnosis of FIRES, an identified syndrome within NORSE, requires a prior febrile infection starting between 2 weeks and 24 h before RSE onset (with or without fever at SE onset) (4, 5). NORSE is a rare disorder (4). In Germany, the annual reported incidence and prevalence of FIRES in pediatric age are estimated to be 1:1,000,000 and 1:100,000, respectively (6). Patients presenting with NORSE or FIRES usually have a very poor prognosis, with mortality rates of 12%−27% and severe neurological sequelae, including cognitive impairment, functional disability, and drug resistant epilepsy in most survivors (7–9).

NORSE etiologies include viral or autoimmune causes. Cases with no identified cause after extensive evaluation are considered as “cryptogenic NORSE” or “NORSE of unknown etiology” (5).

So far, there is currently no high evidence to guide NORSE and FIRES treatment since most therapeutic approaches come from expert opinions and few case reports.

The ketogenic diet (KD) is an established, effective non-pharmacological treatment for drug-resistant epilepsy (10), and in the last decade, an increasing number of studies reported on the efficacy and tolerability of KD in intensive care units (ICU) as an emerging treatment option for SRSE (7, 11–13).

We reported our experience at a pediatric single tertiary center on the use of KD in patients with SRSE, specifically assessing the response in those with NORSE presentation. Our results were combined with the evidence provided by a systematic review of the literature. Finally, we aimed to evaluate the effectiveness of KD in patients presenting with SRSE and NORSE, using time to treatment response as the outcome measure, and to assess the impact of NORSE related characteristics on KD efficacy.

## 2. Methods

### 2.1. Study population

A retrospective review of the medical records was performed at the Necker Enfants Malades Hospital from April 2010 to October 2020. All children with SRSE in whom KD was started as adjunctive therapy were included. For each participant, we recorded and analyzed the following variables: age at SRSE onset, gender, previous history of epilepsy, SRSE etiology, number of treatments prior to KD, time lapse from SRSE onset to KD initiation, fasting at KD initiation, KD ratio, time to achieve ketosis from KD initiation, KD efficacy to stop SRSE, time to SRSE resolution after KD initiation, length of KD, side effects, number of antiseizure medications (ASMs) at hospital discharge, time of follow-up, and outcomes. We identified patients with NORSE presentation, specifying those with FIRES or with NORSE with unknown etiology.

### 2.2. Search strategy and study selection

A systematic review was performed in the electronic databases MEDLINE (PubMed), EMBASE, and Cochrane Library, with the following search terms: “ketogenic” AND (“refractory status epilepticus” OR “super refractory status epilepticus” OR “intensive care unit” OR “new onset refractory status epilepticus” OR “NORSE” OR “febrile infection related epilepsy syndrome” OR “FIRES”).

The relevant studies have been selected with no date restriction, including children and adult patients. The reference lists of the included articles were also searched manually to find any additional eligible papers. The search was up to date as for the 2nd October 2022.

The results of this systematic review were reported according to the recommendations of the Preferred Reporting Items for Systematic Reviews and Meta-Analyses (PRISMA) statement (14).

All study designs with individual details, including at least one patient of any age with SRSE in whom KD was started, have been included. Duplicate records were excluded. Reviews, meta-analyses, editorials, commentaries, and expert opinions were excluded. Titles and abstracts were screened for study eligibility, and full-text articles were reviewed by SM and PDL. Any disagreement was resolved by discussion with a third review author (RN).

For each selected study, the following data were extracted on individual bases when available: age at SRSE onset, gender, previous history of epilepsy, etiology of SRSE, number of treatments (ASMs and anesthetic agents) prior to KD start, other treatments (i.e., steroids) prior to start KD, the time lag from SRSE onset to KD initiation, fasting at KD initiation, KD ratio, time to achieve ketosis, KD efficacy to stop SRSE, time to SRSE resolution after KD initiation, length of KD, side effects, number of treatments at hospital discharge, time of follow-up, and outcomes. Patients with NORSE presentation were selected, specifying those with FIRES or with NORSE with unknown etiology.

### 2.3. Data analysis

Demographic and SE characteristics were summarized by standard descriptive measures.

The primary outcome was the responder rate, defined as clinical and electroencephalographic (EEG) resolution. Kaplan–Meier survival curves were generated for the time-to-KD response. As secondary outcomes, Cox proportional hazard models were created to assess the impact of the following factors on KD efficacy: age at SRSE onset, gender, previous history of epilepsy, etiology, the clinical presentation with NORSE/FIRES, number of treatments prior to KD, the time lag from SRSE onset to KD initiation, fasting at KD initiation, KD ratio, time to achieve ketosis from KD initiation, and side effects. A *p*-value ≤ 0.05 was considered statistically significant. Data were analyzed using STATA/IC version 15 (StataCorp LLC, College Station, TX, USA).

## 3. Results

### 3.1. Single center experience

Overall, 16 children (six female) receive KD for treatment of SRSE at the Necker Enfants Malades Hospital. The median age at SRSE onset was 2 years old (IQR: 1–3, range: 1 month−10 years). Before admission for SRSE, 9/16 (56.2%) had a history of epilepsy. SRSE was due to defined epileptic syndromes in six patients (37.5%), and 6/16 (37.5%) had a progressive symptomatic cause. One patient had acute symptomatic etiology of SRSE due to cerebral anoxia. The remaining presented with NORSE due to infectious encephalitis (*n* = 1) and FIRES (*n* = 2) of unknown etiology. Before KD initiation, they received a median number of ASMs and anesthetics of 4 (IQR: 3–5; range: 2–7), and other treatments, including steroids (*n* = 2), IVIg (*n* = 1), and vitamin therapy (*n* = 2). The median delay from SRSE onset to KD initiation was 2.5 days (IQR: 2–7; range: 1–20).

KD was effective in achieving SRSE cessation in 5/16 (31.25%), after a median time from starting KD of 4.5 days (IQR: 1.5–16; range: 1–30). Side effects due to KD treatment were detected in 7/16 (43.75%), 3/16 died during the acute phase of SRSE, while at hospital discharge, 12/16 (75%) patients had ongoing seizures and received a median number of ASMs of 1 (IQR: 1–3; range: 1–5).

Table 1 summarizes patients' characteristics and details on KD administration, while Table 2 summarizes the response to KD and outcomes.

**Table 1:** Necker Enfants Malades Hospital experience: patients' characteristics.

**Patient**	**Age at SE onset**	**Gender**	**Previous history of epilepsy**	**Etiology**	**Treatments before KD**	**Duration of SE prior KD (days)**	**Fasting at KD initiation**	**KD ratio**	**Time to reach steady ketosis (days)**
1	5 years	F	Yes	SE in defined electroclinical syndrome (DEE)	2 ASMs/anesthetics	4	Yes	4:1	4
2	10 years	M	Yes	Progressive (Alpers syndrome)	3 ASMs/anesthetics	1.5	No	4:1	1
3	3 years	M	No	Progressive (mitochondrial defect)	6 ASMs/anesthetics, IVIg	20	No	4:1	1
4	13 months	M	Yes	Progressive (Chediak Higashi syndrome)	4 ASMs/anesthetics	1.5	No	4:1	1
5	16 months	F	Yes	Progressive (Alpers syndrome)	7 ASMs/anesthetics, steroids	9	No	4:1	4
6	3 years	M	No	Acute (NORSE: infectious encephalitis)	5 ASMs/anesthetics	2	No	4:1	1
7	19 months	M	Yes	SE in defined electroclinical syndrome (Dravet syndrome)	3 ASMs/anesthetics	2	No	4:1	1
8	3 years	M	No	SE in defined electroclinical syndrome (neuro-cutaneous melanosis)	5 ASMs/anesthetics	1	Yes	4:1	1
9	2 years	M	No	Acute (Cerebral anoxia)	2 ASMs/anesthetics	2	No	4:1	1
10	2 years	F	Yes	SE in defined electroclinical syndrome (Dravet syndrome)	5 ASMs/anesthetics	4	No		3
11	4 years	F	No	Unknown (FIRES)	4 ASMs/anesthetics	5	No		1
12	2 months	M	No	Unknown (FIRES)	6 ASMs/anesthetics, vitamins	2	No		0
13	2 months	M	Yes	SE in defined electroclinical syndrome (EIMFS)	4 ASMs/anesthetics	2	Yes		1
14	1 month	F	No	Progressive (mitochondrial defect)	3 ASMs/anesthetics, pyridoxine	3	No		0
15	2 years	M	Yes	SE in defined electroclinical syndrome (Dravet syndrome)	4 ASMs/anesthetics, steroids	11	Yes		2
16	13 months	F	Yes	Progressive (mitochondrial defect)	4 ASMs/anesthetics	13	No		2

ASMs, antiseizure medications; DEE, developmental and epileptic encephalopathy; EIMFS, epilepsy of infancy with migrating focal seizures; FIRES, febrile infection-related epilepsy; Syndrome KD, ketogenic diet; NORSE, now onset refractory status epilepticus; SE, status epilepticus.

**Table 2:** Necker Enfants Malades Hospital experience: response to KD and outcome.

**Patient**	**KD efficacy to stop SE**	**Time to SE resolution (days)**	**Total length of KD (days)**	**Side effects**	**No. ASMs at discharge**	**Outcome**
1	Yes	1	570	None	4	Ongoing seizures
2	Yes	1.5	1,500	None	2	Ongoing seizures
3	No	20	16	None	/	Dead
4	Yes	1.5	7	Vomiting	2	Ongoing seizures
5	No	9	16	Hypoglycemia	4	Ongoing seizures
6	No	2	12	None	/	Dead
7	No	2	4	Hypoglycemia	1	Ongoing seizures
8	Yes	1	9	None	2	Ongoing seizures
9	Yes	2	3	None	1	Ongoing seizures
10	No	4	16	Hypoglycemia	/	Dead
11	No	5	8	Hypoglycemia	2	Ongoing seizures
12	No	2	5	None	1	Ongoing seizures
13	No	2	3	Hypoglycemia	5	Ongoing seizures
14	No	3	10	None	1	Ongoing seizures
15	No	11	20	None	2	Ongoing seizures
16	No	13	90	Weight loss	3	Ongoing seizures

ASMs, antiseizure medications; KD, ketogenic diet; SE, status epilepticus.

### 3.2. Literature systematic review

**Figure 1:**
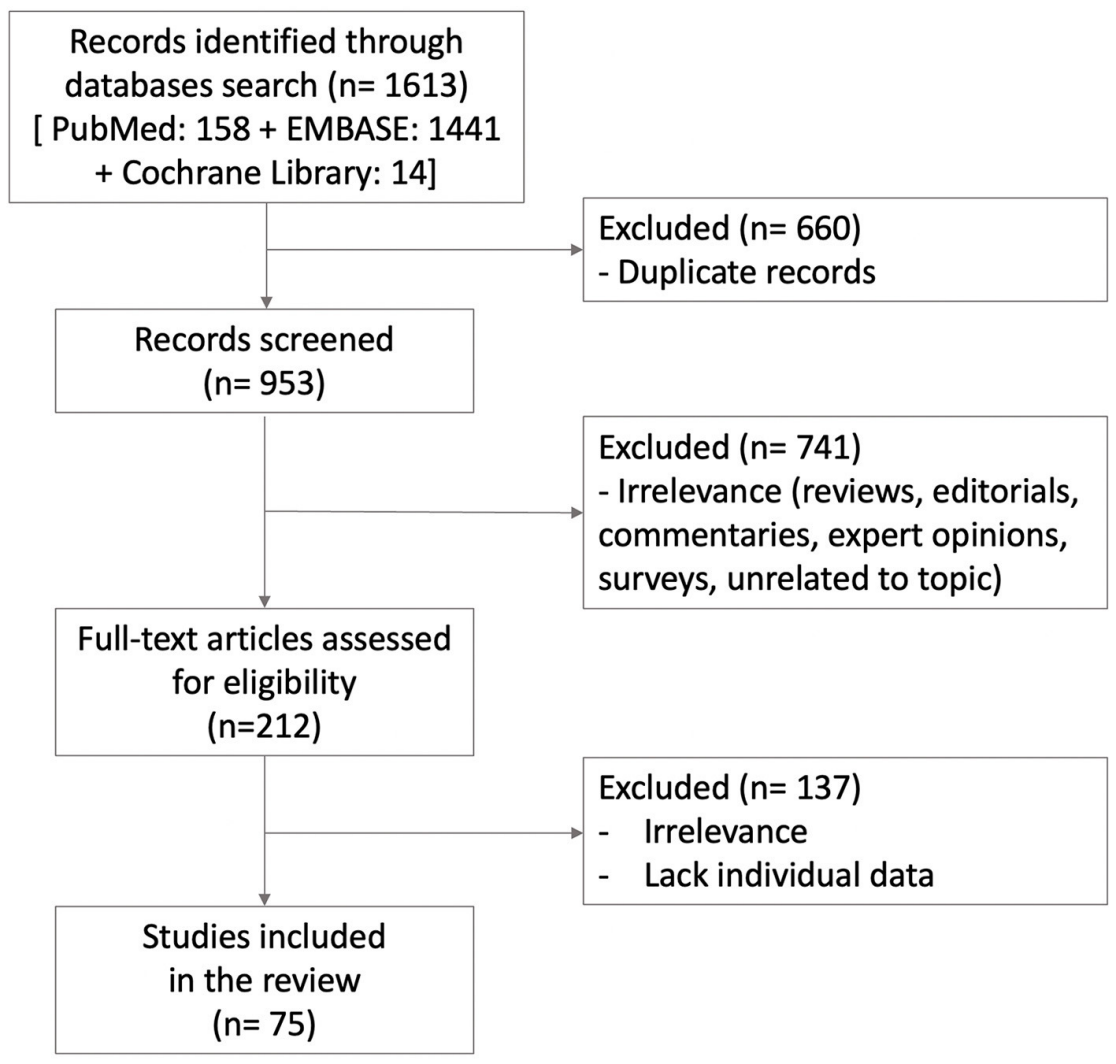
Flow diagram of the study selection process.

One thousand six hundred thirteen records were initially identified. Two hundred and twelve were retrieved for detailed assessment, of which 75 were included in the review (Figure 1). The selected studies were retrospective observational studies (*n* = 21) (7, 11, 13, 15–32), single cases (*n* = 43) (33–75), and small case series (*n* = 10) (76–85); only one study is a prospective, open-label, single-arm observational study (86). There were no randomized or non-randomized clinical trials. All included studies were considered to have a high risk of bias related to the retrospective study design, patient selection and data collection, ascertainment bias, missing data, and reporting of the results.

Table 3 summarizes patients' characteristics and details on KD administration and Table 4 summarizes the response to KD and outcomes.

**Table 3:** Systematic review: summary of patients' characteristics and KD administration.

**References**	**Study design**	**Population**	**Age at SE onset (years)**	**Gender**	**Previous history of epilepsy**	**Etiology**	**No. of ASMs & anesthetics before KD**	**Other treatment before KD**	**Duration of SE prior KD (days)**	**Fasting at KD initiation**	**KD ratio**	**Time to reach steady ketosis (days)**
Aydemir and Kandula (33)	Case report	1 adult	27	M	0%	Unknown 100% (NORSE)	10	Immunotherapy, Electroconvulsive therapy	16	–	–	–
Chomtho et al. (15)	Retrospective observational	14 children	Median 7 (IQR 8 months−9 years, range 2 months−13.6 years)	F 50%, M 50%	35.7%	Acute 28.5% (NORSE: HSV and Rickettsia encephalitis, anti-NMDAR encephalitis); SE in defined electroclinical syndrome 35.7% (PLP deficiency, LGS, epilepsy due to focal cortical dysplasia); progressive 7.3% (RE); unknown 28.5% (NORSE/FIRES)	Median 6 (IQR 5–8, range 3–9)	Steroids 64.3%, IVIg 42.8%, pyridoxine 71.4%, PLP 35.7%, PLEX 7.1%, cyclophosphamide 7.1%, hypothermia 7.1%, epilepsy surgery 14.3%	Median 6 (IQR 5–9, range 4–14)	–	variable	Median 3.5 (IQR 2–7, range 1–9)
Dutta et al. (34)	Case report	1 adult	35	F	0%	Acute (hypoxic brain injury)	8	No	14	–	MCTKD	3
Giménez-Roca et al. (35)	Case report	1 adult	39	F	100%	SE in defined electroclinical syndrome (IGE)	10	MPN, IVIg	30	–	–	–
Luo et al. (36)	Case report	1 child	2.3	M	0%	Unknown (FIRES)	7	No	17	–	3.1	–
Orlandi et al. (37)	Case report	1 Adult	38	F	0%	Unknown (NORSE)	12	Allopregnanolone, magnesium sulfate, hypothermy, PLEX, IVIg, MPN	133	–	–	–
Perulli et al. (38)	Case report	1 child	11	F	0%	Unknown (FIRES)	12	MPN, IVIg, PLEX	32	–	–	7
Sivathanu et al. (39)	Case report	1 child	7	M	0%	Acute (NORSE in anti-GAD 65 encephalitis)	7	IVIg, MPN, RTX	9	–	4:1	2
Varughese et al. (40)	Case report	1 child	0.6	F	100%	SE in defined electroclinical syndrome (PCDH19)	3	–	3	–	–	–
Allen et al. (41)	Case report	1 adult	19	M	100%	SE in defined electroclinical syndrome (UBE2A deficiency syndrome)	11	Steroids	81	–	–	7
Anand et al. (76)	Case series	3 adults and 1 child	Median 25.5 (IQR 15.5–43.5, range 7–60)	F 25%, M 75%	50%	Acute 25% (stroke); SE in defined electroclinical syndrome 25% (LGS); remote 25% (post-encephalitis); unknown (NORSE) 25%	Median 6 (IQR 5–6.5, range 4–7)	Steroids (12.5%)	Median 3 (IQR 2–14, range 2–14)	–	4:1 (75%)	Median 3 (IQR 2–4.5, range 2–5)
Baba et al. (42)	Case report	1 child	8	F	0%	Unknown 100% (FIRES)	6	Steroids, IVIg	6	–	4:1	3
Breu et al. (16)	Retrospective observational	8 children	Median 1.12 (IQR 0.08–6.88, range 0.03–12.28)	F 50%, M 50%	62.5%	SE in defined electroclinical syndrome 50% (Ohtahara syndrome, IS due to SCN2A pathogenic variant, TSC); progressive 37.5% (Alpers syndrome); Unknown 12.5% (FIRES)	Median 5 (IQR 2.5–7, range 1–7)	Steroids (12.5%)	Median 6 (IQR 1.5–9, range 1–42)	–	4:1	Median 2.8 (IQR 1.14–8.52, range 1–17.9)
Camões et al. (77)	Case series	3 adults	Median 20 (IQR 20–38, range 20–38)	F 66.6%, M 33.3%	0%	Acute 33.3% (head trauma), unknown 66.6% (NORSE)	–	–	Median 5 (IQR 4–9, range 4–9)	Yes (48 h)	4:1 (100%)	Median 4.5 (IQR 1–8, range 1–8)
Donnelly et al. (43)	Case report	1 adult	26	F	0%	Unknown (NORSE)	14	MPN, RTX, L salpingo-oophorectomy, hypothermia, electroconvulsie therapy, PLEX, pyridoxine	56	–	–	–
Katz et al. (44)	Case report	1 adult	29	F	0%	Unknown 100% (NORSE)	20	Steroids, IVIg, PEX, CYC, empiric bilateral partial oophorectomy	28	–	5:1	9
Kaul et al. (45)	Case report	1 adult	65	M	0%	Acute (subarachoid hemorrhage)	5	No	25	–	2.3:1	4
Schoeler et al. (13)	Retrospective observational	8 children	Median 7 (IQR 6.6–9.6, range 5.8–10.8)	F 25%, M 75%	0%	Unknown 100% (FIRES)	Median 9 (IQR 8–14, range 8–16)	Immunotherapy	Median 13 (IQR 11.5–15, range 6–24)	–	4:1 (62.5%), 5:1 (12.5%), 3.1 (25%)	Median 3 (IQR 2–7.5, range 1–12)
Aurangzeb et al. (46)	Case report	1 adult	22	M	0%	Unknown 100% (NORSE)	9	Immunotherapy	27	–	–	0
Chee et al. (47)	Case report	1 child	14	F	0%	Unknown 100% (FIRES)	9	Steroids, IVIg, hypothermia, tocilizumab	–	–	–	–
Chiu and Datta (48)	Case report	1 child	11	M	0%	Acute 100% (Childhood primary angiitis of the CNS)	6	Steroids, IVIg, cyclophosphamide	24	–	4:1	–
Gupta et al. (49)	Case report	1 child	0.3	F	100%	SE in defined electroclinical syndrome 100% (Ohtahara syndrome due to AIMP1 pathogenic variant)	12	–	4	–	4:1 (100%)	2
Koessler et al. (50)	Case report	1 child	16	F	0%	Progressive 100% (Alpers syndrome)	9	Steroids, IVIg	9	–	4:1 (100%)	5
Noviawaty et al. (51)	Case report	1 adult	38	M	0%	Unknown 100% (NORSE)	9	Steroids	49	–	4:1 (100%)	5
Vallecoccia et al. (52)	Case report	1 adult	34	M	0%	Unknown 100% (NORSE)	10	Steroids, PEX, IVIg, tocilizumab	49	–	–	–
Wang et al. (17)	Retrospective observational	10 children	Median 9 (IQR 7–10, range 5–13)	F 60%, M 40%	0%	Unknown 100% (FIRES)	Median 3 (IQR 3–4, range 2–4)	–	Median 9 (IQR 6–20, range 2–22)	3 days	4:1 (100%)	Median 6 (IQR 3–10, range 1–14)
Arayakarnkul and Chomtho (18)	Retrospective observational	13 children	Median 8.3 (IQR 1.7–9.8, range 0.2–13.5)	F 46.1%, M 53.8%	30.8%	Acute 37.5% (intracranial hemorrhage, NORSE: infectious encephalitis, autoimmune encephalitis); progressive 7.7% (RE), SE in defined electroclinical syndromes 23.1% (PLP deficiency, LGS), unknown 23% (NORSE/FIRES)	Median 8 (IQR 7–9, range 5–12)	Steroids 69.2%, pyridoxine 84,6%, PLP 53.8%, IVIg 46.1%, hypothermia7.7%, PEX 7.7%	–	12 h	–	Median 2 (range 1.3–4.6)
Dilena et al. (53)	Case report	1 child	10	M	0%	Unknown (FIRES)	10	Steroids, IVIg, Mg	–	–	–	–
Francis et al. (19)	Retrospective observational	11 adults	Median 46 (IQR 31–72, range 21–73)	F 45.4%, M 54.5%	45.4%	Acute 63.6% (NORSE: anti-NMDAR encephalitis; intracranial hemorrhage, cardiac arrest, stroke, intracranial hemorrhage, ethanol withdrawal); remote 27.3% (traumatic brain injury sequelae); SE in defined electroclinical syndromes 9%	Median 3 (IQR 2–3, range 2–8)	–	Median 1 (IQR 0–2, range 0–3)	–	–	Median 1 (IQR 0–2, range 0–5)
Park et al. (20)	Retrospective observational	14 children, 2 adults	Median 8 (IQR 5–13.5, range 0.1–40)	F 37.5%, M 62.5%	12.5%	Acute 12.5% (NORSE: HSV encephalitis, enteroviral encephalitis), remote 12.5% (hypoxic ischemic encephalopathy), SE in defined electroclinical syndromes 12.5% (FLE, hemimegalencephaly), Unknown 62.5% (FIRES)	5 ASMs (range 2–8); 2 anesthetics (range 1–3)	None	Median 23 (IQR 12–33.5, range 3–420)	–	–	–
Peng et al. (21)	Retrospective observational	7 children	–	F 57%, M 43%	0%	Unknown 100% (FIRES)	Median 6 (IQR 5–7, range 5–7)	Steroids 71.4%, IVIg 100%, PEX 57%	Median 11 (IQR 4–15, range 3–31)	None	4:1 (42.8%), 3:1 (28.6%), 3:2 (14.3%), 2:5 (14.3%)	Median 3 (IQR 1–5, range 1–11)
Arya et al. (22)	Retrospective observational	14 children	–	–	14.2%	Acute 7.1% (NORSE: possible autoimmune encephalitis), remote 7.1% (MCD), SE in defined electroclinical syndromes 7.1% (TSC); unknown 78.5% (NORSE/FIRES)	–	Steroids 14.2%, IVIg 14.2%, PEX 21.4%, pyridoxine 7,1%, VNS 14.2%	Median 13 (IQR 5–18, range 3–39)	–	4:1 (78.6%), 3:1 (7.1%), 3:5 (7.1%), 5:1 (7.1%),	Median 2 (IQR 1–3, range 1–10)
Blunck et al. (54)	Case report	1 adult	42	F	100%	Remote (cerebral palsy)	11	SGLT2 inhibitor	–	24 h	4:1	20
Lee and Chi (23)	Retrospective observational	7 children	Median 11.2 (IQR 5.7–11.7, range 1.2–17.8)	F 71.4%, M 28.5%	0%	Unknown 100% (FIRES)	**–**	Steroids 28.5%	–	–	–	–
Cervenka et al. (86)	Prospective observational	15 adults	Median 47 (IQR 25–63, range 18–82)	F 66.6%; M 33.3	40%	Acute 40% (anoxic ischemic injury, intracranial hemorrhage, encephalitis, haemorrhagic infarct); progressive 6.6% (glioblastoma multiforme); SE in defined electroclinical syndromes 20% (LGS, focal epilepsy); unknown 33.3% (NORSE)	Median 8 (IQR 6–9, range 5–12)	Steroids 26.6%, PEX 33.3%, CYC 6.6%	Median 10 (IQR 5–19, range 2–39)	24 h (20%)	4:1 (100%)	Median 2 (IQR 1–7, range 1–16)
Farias-Moeller et al. (24)	Retrospective observational	9 children	Median 5 (IQR 5–8, range 2–8)	F 66.6%, M 33.3%	11.1%	Progressive 11.1% (CNS hemophagocytic lymphoistiocytosis), SE in defined electroclinical syndrome 11.1%; Unknown 77.8% (FIRES),	Median ASMs 4 (IQR 3–4); median anesthetic agents 2 (IQR 2–3)	Steroids 88.8%	Median 13 (IQR 10–16, range 7–41)	8 h (11.1%)	4:1 (77.7%), 3.1 (11.1%), 2.75:1 (11.1%)	Median 3 (IQR 3–4, range 2–13)
Fox et al. (55)	Case report	1 child	6	F	0%	Acute (FIRES)	8	Steroids, IVIg, PEX, biotin, folinic acid, pyridoxine, L-carnitine	–	–	–	–
Uchida et al. (56)	Case report	1 adult	20	F	0%	Acute (NORSE: anti-NMDAR encephalitis)	7	Steroids; IVIg, ovariectomy	–	–	–	–
Appavu et al. (57)	Case report	1 child	8 months	F	100%	SE in defined electroclinical syndrome (DEE due to TBC1D24 pathogenic variant)	3	None	–	–	–	–
Appavu et al. (57)	Retrospective observational	10 children	Median 8 (IQR 3.5–15, range 2–16)	F 40%, M 60%	40%	Acute 40% (PCDH19, GABRG2, anti-NMDAR encephalitis, mycoplasma post-infectious encephalitis), progressive 10% (RE), SE in defined electroclinical syndrome 30% (LGS, DR GE, non-ketotic hyperglycinemia), unknown 20% (NORSE/FIRES)	Median 5.5 (IQR 4–6, range 3–8)	MPN, IVIg, PLEX, ACTH 40%	Median 18 (IQR 8–28, range 1–45)	–	4:1 (90%), 5:1 (10%)	Median 5.5 (IQR 2–8, range 1–13)
Chiusolo et al. (58)	Case report	1 child	8	M	100%	SE in defined electroclinical syndrome (GE and ASD)	8	Clozapine, steroids, IVIg	–	–	3:1	3
Kenney-Jung et al. (59)	Case report	1 child	32 months	F	0%	Unknown (FIRES)	8	Steroids	6	–	4:1	47
Mirás Veiga et al. (60)	Case report	1 child	4	M	0%	Unknown (FIRES)	6	MPN, IVIg	4	–	–	–
Amer et al. (61)	Case report	1 adult	21	F	0%	Acute (NORSE: anti-NMDAR-encephalitis)	6	Steroids, IVIg, PEX	–	–	4:1	–
Caraballo et al. (78)	Case series	2 children	23 and 17 months	M 100%	100%	Progressive 50% (PME), SE in defined electroclinical syndrome 50% (myoclonic epilepsy)	5 and 4	Steroids 100%	15 and 21	24 h (100%)	4:1	–
Cash (62)	Case report	1 adult	50	M	0%	Remote (hypoxic ischaemic brain injury)	10	None	36	–	4:1	–
Cobo et al. (79)	Case series	4 children	Median 8.5 (IQR 3–12, range 0.16–13)	F 25%, M 75%	50%	SE in defined electroclinical syndrome 50% (EIMFS, TSC), unknown 50% (NORSE)	7 (range 7–8)	Pyridoxine, pyridoxal-5-phosphate, folinic acid 25%; IVIg 25%	Median 28 (IQR 19.5–51.5, range 19–67)	0%	4:1 (50%), 3:1 (25%), 2:1 (25%)	Median 5
Fung et al. (80)	Case series	4 children	Median 12 (IQR 7–16, range 6–16)	F 50%, M 50%	0%	Acute 50% (VGKC associated encephalitis, possible autoimmune encephalitis), unknown 50% (FIRES)	Median 6.5 (IQR 6–7, range 6–7)	PLEX 50%, vitamin B6 and folinic acid 50%	Median 17.5 (IQR 14.5–19.5, range 12–21)	–	4:1 (100%)	–
Incecik et al. (63)	Case report	1 child	16	F	100%	Remote (cerebral palsy)	8	Steroids, IVIg	–	–	–	–
Lin et al. (64)	Case report	1 child	6.3	M	0%	Unknown (NORSE)	6	None	2	0%	4:1	1
Moriyama et al. (65)	Case report	1 child	9	F	0%	unknown (FIRES)	4	None	15	0%	3:1	1
Barros et al. (66)	Case report	1 child	7	M	0%	Acute (NORSE: anti-NMDAR-encephalitis)	12	MPN, IVIg, PLEX, RTX	–	–	–	–
Caraballo et al. (26)	Retrospective observational	10 children	Median 8 (IQR 5–10, range 0.5–16)	F 40%, M 60%	0%	Unknown 100% (NORSE/FIRES)	3	IVIg 100%	–	100%	4:1	3
Fung (67)	Case report	1 child	16	F	0%	Acute (NORSE: encephalitis)	3	None	18	–	4:1	–
Gedik et al. (68)	Case report	1 child	5	M	0%	Acute (NORSE: meningoencephalitis)	11	IVIg	27	–	–	–
Matsuzono et al. (69)	Case report	1 adult	22	M	0%	Unknown (FIRES)	9	Steroids	155	–	–	–
O'Connor et al. (27)	Retrospective observational	5 children	Median 9 (IQR 5–9, range 0.83–10)	F 40%, M 60%	40%	Progressive 40% (Alpers syndrome, mitochondrial defect), SE in defined electroclinical syndrome 20% (GE), Unknown 40% (NORSE/FIRES)	Median 8 (IQR 5–9, range 5–9)	IVIg 40%	Median 10.5 (IQR 4.5–18, range 3–21)	0%	4:1	Median 5 (IQR 2–5, range 1.5–8)
Singh et al. (81)	Case series	2 children	7 and 10	F 50%, M 50%	0%	Unknown 100% (FIRES)	7	Steroids 100%	13 and 3	No	4:1 and 6:1	2 and 20
Thakur et al. (28)	Retrospective observational	10 adults	Median 33.5 (IQR 28–48, range 23–51)	F 60%, M 40%	10%	Acute 90% (anoxic ischemic injury, NORSE: infectious encephalitis, autoimmune encephalitis), remote 10% (cortical dysplasia)	Median 7.5 (IQR 5–12, range 5–13)	Steroids 50%	Median 21.5 (IQR 17–45, range 2–60)	70%	4:1 (90%), 3:1 (10%)	Median 3 (IQR 1–6, range 0.5–7)
Caraballo et al. (82)	Case series	2 children	12 and 9.5		0%	Unknown 100% (FIRES)	2 and 5	Immunotherapy 50%	–	–	–	–
Sort et al. (83)	Case series	3 children	Median 10 (range 3–11)	F 33.3%, M 66.6%	33.3%	Progressive 33.3% (mitochondrial defects), unknown 66.6% (HHE, FIRES)	Median 8 (range 6–9)	Steroids 66.6%, IVIg 33.3%, hypothermia 33.3%, PLEX 33.3%	Median 7 (range 5–47)	–	5:1 (33.3%)	Median 12 (range 1–17)
Strzelczyk et al. (70)	Case report	1 adult	21	F	100%	Progressive (Lafora disease)	8	Steroids, magnesium	15	–	4:1	3.5
Martikainen et al. (71)	Case report	1 adult	26	F	0%	Progressive (Alpers syndrome)	3	–	7	–	LGIT (low glycemic index treatment)	–
Vaccarezza et al. (29)	Retrospective observational	5 children	Median 6 (IQR 4–12, range 1–14)	F 66.6%, M 33.3%	0%	SE in defined electroclinical syndrome 20% (DR structural FE), unknown 80% (HHE, FIRES)	Median 7 (IQR 7–8, range 5–8)	Steroids 20%, IVIg 60%	Median 30 (IQR 18–45, range 15–52)	100%	4:1	2.5
Cervenka et al. (72)	Case report	1 adult	49	M	0%	Acute (NORSE: cerebral inflammation)	12	PLEX, epilepsy surgery	57	–	4:1	11
Ismail and Kossoff (2011)	Case report	1 child	14	F	0%	Unknown (FIRES)	10	None	60	–	4:1	2
Kramer et al. (7)	Retrospective observational	7 children	Median 6 (IQR 5–9, range 4–9)		0%	Unknown 100% (FIRES)	Median 6 (IQR 4–8, range 2–13)	Steroids 42.8%, IVIg 71.4%, PLEX 14.3%, vitamin B6 14.3%, Folinic acid 14.3%	–	–	–	–
Nam et al. (30)	Retrospective observational	4 children, 1 adult	Median 10 (IQR 8–14, range 4–40)	F 60%, M 40%	0%	Acute 100% (NORSE: infectious encephalitis)	Median 8 (IQR 8–10, range 5–11)	None	Median 30 (IQR 30–120, range 15–420)	–	4:1	
Kumada et al. (84)	Case series	2 children	3 and 5	F 100%	100%	SE in defined electroclinical syndrome 50% (FLE), remote 50% (subcortical band heterotopia)	7 and 3	–	390 and 150	–	4:1	3
Nabbout et al. (31)	Retrospective observational	9 children	Median 6 (IQR 5–7, range 4–8)	F 55.5%, M 44.4%	0%	Unknown 100% (FIRES)	Median 5 (IQR 4–6, range 3–7)	Steroids 77.8%	Median 17 (IQR 8–30, range 4–55)	100%	4:1	Median 3 (IQR 2–3, range 0–4)
Wusthoff et al. (85)	Case series	2 adults	34 and 29	F 50%, M 50%	50%	Acute 50% (NORSE: infectious encephalitis), progressive 50% (RE)	8 and 10	IVIg 50%, steroids 50%	20 and 101	50%	4:1	8 and 10
Villeneuve et al. (11)	Retrospective observational	4 children, 1 adult	Median 8 (IQR 3–10, range 1–18)	F 60%, M 40%	100%	Acute 20% (NORSE: infectious encephalitis), remote 20% (Sturge Weber syndrome), progressive 20% (Ito syndrome), SE in defined electroclinical syndrome 40% (cryptogenic epilepsy)	Mean 2.7	None	–	100%	4:1	–
Bodenant et al. (74)	Case report	1 adult	54	M	100%	SE in defined electroclinical syndrome (FE)	10	–	31	–	4:1	–
Baumeister et al. (75)	Case report	1 child	10	M	100%	SE in defined electroclinical syndrome (FE)	6	–	–	48 h	4:1	2
François et al. (32)	Retrospective observational	6 children	–	–	0%	Remote 100% (postencephalitic)	Mean 6 (range 3–10)	–	Median 11 (IQR 8–75, range 8–75)	100%	3.1/4.1	2

ACTH, adrenocorticotropic hormone; ASMs, antiseizure medications; CNS, central nervous system; CYC, cyclophosphamide; DEE, developmental and epileptic encephalopathy; DR, drug resistant; EIMFS, epilepsy of infancy with migrating focal seizures; FE, focal epilepsy; FIRES, febrile infection-related epilepsy syndrome; FLE, frontal lobe epilepsy; GAD, antiglutamic acid decarboxylase; HHE, Hemiconvulsion-Hemiplegia-Epilepsy syndrome; HSV, herpes simplex virus; GE, generalized epilepsy; IGE, idiopathic generalized epilepsy; IQR, interquartile range; IVIg, intravenous immunoglobulins; KD, ketogenic diet; L, left; LGS, Lennox-Gastaut syndrome; MCD, malformations of cortical development; Mg, magnesium; MPN, methylprednisolone; NMDAR, N-methyl-D-aspartate receptor; No, number; NORSE, new onset refractory status epilepticus; PLEX, plasma exchange; PLP, pyridoxal-5-phosphate; PME, progressive myoclonic epilepsy; RE, rasmussen encephalitis; RTX, Rituximab; SE, status epilepticus; TSC, tuberous sclerosis complex; VCKC, voltage-gated potassium channel.

**Table 4:** Systematic review: response to KD and outcomes.

**References**	**KD efficacy to stop SE**	**Time to SE resolution (days)**	**Total length of KD (days)**	**Side effects**	**No. ASMs at discharge**	**Follow-up (months)**	**Outcomes**
Aydemir and Kandula (33)	0%	90	24	None	6	2	Baseline functional status
Chomtho et al. (15)	92.9%	Median 11 (IQR 7–14, range 4–17)	–	Electrolyte imbalance (85.7%), hypercalciuria (71.4%), hypertriglyceridemia (64.3%), hypoglycemia (21.4%)	–	–	Seizure free 85.7%, Dead 14.3%
Dutta et al. (34)	100%	10	90	None	–	3	Neurorehabilitation
Giménez-Roca et al. (35)	100%	32	–	None	–	–	Baseline functional status
Luo et al. (36)	0%	42	36	None	4	1	Mild DD
Orlandi et al. (37)	0%	187	13	Elevation of liver and pancreatic enzymes	4	42	Severe ID, tetraparesis, DR epilepsy
Perulli et al. (38)	100% (with Anakinra)	48	105	None	3	3	Moderate ID
Sivathanu et al. (39)	100%	3	90	None	3	12	Mild delay
Varughese et al. (40)	0%	52	49	None	5	6	Mild delay
Allen et al. (41)	100%	7	–	None	3	24	–
Anand et al. (76)	100%	Median 6 (IQR 3.5–8, range 2–9)	Median 31 (IQR 24–166, range 18–300)	None	Median 2 (IQR 2–2.5, range 2–3)	Median 1 (IQR 1–10, range 1–10)	–
Baba et al. (42)	100%	–	15	Elevated liver and pancreatic enzymes	2	15	Neurological sequelae
Breu et al. (16)	75%	Median 1.5 (IQR 1–5, range 1–15)	–	Dehydration (12.5%), dystrophia (12.5%), constipation (25%), flatulence (12.5%), hypertriglyceridemia (25%), hyperlipasemia (12.5%), high ketosis (12.5%), diarrhea (25%), pancreatitis (12.5%), catecholamines (12.5%), hepatopathy, hypercholesterinemia (12.5%), reduced drinking (12–5%), weight loss (12.5%), paralytic ileus (12.5%)	–	Median 5 (IQR 3–12, range 3–12)	Dead (62.5%), seizure free after epilepsy surgery (12.5%), daily seizures (12.5%), monthly seizures (12.5%)
Camões et al. (77)	66.6%	Median 14 (IQR 13–15, range 13–15)	Median 32 (IQR 11–41, range 11–41)	Hypoglycemia (66.6%), gastric statis (33.3%), hypertriglyceridemia (33.3%), ileus (33.3%), septic shock (33.3%)	–	–	Seizure freedom (66.6%), dead (33.3%)
Donnelly et al. (43)	0%	84	14	Elevated liver enzymes	2	2	Mild ID
Katz et al. (44)	100%		45	None	–	7	cardiac arrest, relapse of SE
Kaul et al. (45)	100%	29	14	None	–	1	Neurorehabilitation
Schoeler et al. (13)	62.5%	Median 19 (IQR 12–31, range 12–35)	Median 21.5 (IQR 16.5–68.5, range 12–383)	Loose stool (62.5%), hyperketosis (37.5%), weight loss (12.5%), elevated amylase and lipase (25%), elevated lactate dehydrogenase (12.5%), hypoglycemia (12.5%), metabolic acidosis (50%), hypertriglyceridemia (37.5%)	–	–	Dead (37.5%), daily seizures and severe ID (25%), weekly-monthly seizure and learning difficulties (25%)
Aurangzeb et al. (46)	0%	–	3	None	–	10	mRS 3, ongoing focal seizures
Chee et al. (47)	0%	–		–	4	4	Seizure freedom, mild neuropsychological impairment
Chiu and Datta (48)	100%	–	86	–	5	18	Seizure freedom, mild neuropsychological impairment
Gupta et al. (49)	100%	10	–	None	–	–	–
Koessler et al. (50)	100%	7	60	Elevated liver enzymes (100%)	2	3	Death after 3 months
Noviawaty et al. (51)	100%	2	68	None	7	12	Ongoing seizures, severe ID
Vallecoccia et al. (52)	0%	–	7	Intolerance and high gastric residual volume (100%)	–	–	–
Wang et al. (17)	80%	Median 8 (IQR 7–15, range 2–30)	Median 165 (IQR 36–240, range 8–365)	Arrhythmia (10%), urinary stones (30%), hematuria (10%)	Median 4 (IQR 3–4, range 0–5)	–	Ongoing seizures and ID (90%)
Arayakarnkul and Chomtho (18)	92.3%	Median 9 (IQR 6.5–11.5, range 6–16)	–	–	–	Median 83 (IQR 57–96, range 15–231)	Dead 15.4%, epilepsy surgery 7.7%, seizure free 77%
Dilena et al. (53)	0%	–	21	–	5	36	Severe ID, seizure improvement with Anakinra
Francis et al. (19)	100%	Median 5 (IQR 2–9, range 2–15)	–	Metabolic acidosis 63.6%, hypoglycemia 18.2%, bowel perforation 9%, infection 9%, elevated liver enzymes 9%, hyponatremia 9%	Median 3 (IQR 3–6, range 2–10)	–	Neurological sequelae 82%
Park et al. (20)	56.2%	6.5 (range 1–28)	Median 61.5 (IQR 30–82.5, range 4–474)	Regurgitation 25%, constipation 12.5%, hypertriglyceridemia 12.5%, aspiration pneumonia 37.5%, nausea 6.2%, vomiting 12.5%, kidney stones 6.2%, metabolic acidosis 6.2%, hypoproteinemia 12.5%, elevated liver enzymes 6.2%	–	–	Ongoing seizures (62.5%), severe ID (18.7%), moderate ID (12.5%), mild ID (50%)
Peng et al. (21)	85.7%	Median 5.5 (IQR 4–6, range 1–10)	Median 90 (IQR 60–90, range 60–330)	Diarrhea (57%), hyperlipidemia (57%), transient hyperamylasemia (14.3%)	Median 4 (IQR 3–4, range 3–4)	Median 14 (IQR 11–31, range 4–40)	Seizure freedom (28.6%), ongoing seizures (57%)
Arya et al. (22)	85.7%	Median 7 (range 7–14)	–	Bowel disturbances 7.1%, Weight loss 7.1%, hypertriglyceridemia 7.1%	Median 5 (IQR 3–5, range 2–7)	–	–
Blunck et al. (54)	0%	–	36	–	8	4	Dead
Lee and Chi (23)	0%	–	–	Elevated liver enzymes 71.4%	Median 4 (IQR 3–5, range 1–5)	Median 31 (IQR 13–74, range 6–89)	Dead 28.5%, ongoing seizures 71.4%, moderate-severe ID 71.4%
Cervenka et al. (86)	73.3%	Median 5 (IQR 3–8, range 0–30)	Median 28 (IQR 15–52, range 4–630)	Hyponatremia 6.6%, constipation 13.3%, metabolic acidosis 26.6%, hyperlipidemia 13.3%, hypoglycemia 13.3%, weight loss 6.6%	–	Median 6 (range 6–21)	Death 33.3%, ongoing seizures 33.3, seizure freedom 20%, lost to FU 13.3
Farias-Moeller et al. (24)	55.5%	Median 7	Median 90 (IQR 30–150, range 7.5–180)	Hypertriglyceridemia 22.2%, pancreatitis 11.1%	Median 3	Median 3	Ongoing seizures 55.5%, cognitive deficits 100%
Fox et al. (55)	–	–	–	None	–	–	Ongoing seizures, severe ID
Uchida et al. (56)	100%	–	–	None	–	–	–
Appavu et al. (57)	100%	–	–	None	–	–	Ongoing seizures
Appavu et al. (57)	90%	Median 8 (IQR 3–15, range 1–30)	–	Ketoacidosis, hypophosphatemia, hypokalemia 10%	Median 3 (IQR 3–4, range 1–5)	Median 12 (IQR 4–29, range 1–39)	Death 10%, ongoing seizures 50%, seizure freedom 30%
Chiusolo et al. (58)	0%	–	8	Elevated liver enzymes	9	4	Ongoing seizures
Kenney-Jung et al. (59)	0%	–	92	–	4	12	Chronic epilepsy
Mirás Veiga et al. (60)	0%	–	–	Liver failure	–	3	Ongoing seizures, cognitive deficits
Amer et al. (61)	100**%**	–	–	–	3	–	–
Caraballo et al. (78)	100%	7	365 and 180	None	1 and 2	12 and 6	Ongoing seizures 50%, lost to FU 50%
Cash (62)	100%	–	–	None	4	3.7	–
Cobo et al. (79)	75%	–	Median 75 (IQR 51–103, range 28–130)	Nephrolithiasis 25%, asymptomatic hypoglycemia 25%, constipation 25%, gastroesophageal reflux 25%	Median 1 (IQR 0.5–2, range 0–3)	Median 2.5 (IQR 1.7–7.7, range 1–13)	Seizure freedom 25%, ongoing seizures 75%
Fung et al. (80)	25%	–	Median 10 (IQR 9.5–10.5, range 9–11)	Hypoproteinemia 25%, vomiting 25%, increase breakthrough seizures 25%	–	Median 2 (IQR 1–4.5, range 1–6)	Refractory epilepsy and cognitive deficits 75%, seizure freedom 25%
Incecik et al. (63)	0%	–	–	–	4	5.5	–
Lin et al. (64)	100%	1.5	90	Weight loss, intermittent diarrhea	5	3	Ongoing seizures
Moriyama et al. (65)	100%	3	26	Protein losing enteropathy		7.4	ongoing seizures, cognitive deficits
Barros et al. (66)	0%	–	–	–	4	24	ongoing seizures, cognitive deficits
Caraballo et al. (26)	70%	6	Median 270 (IQR 60–540, range 7–1080)	Pancreatitis 20%, severe vomiting and hypoglycemia 10%	–	–	–
Fung (67)	0%	–	10	None	–	–	–
Gedik et al. (68)	0%	–	–	–	–	2	Seizure freedom
Matsuzono et al. (69)	100%	25	–	None	–	10	Seizure freedom, cognitive deficits
O'Connor et al. (27)	100%	Median 5 (IQR 2–5, range 2–8)	Median 405 (IQR 360–495, range 360–540)	None	–	Median 13.5 (IQR 12–16.5, range 12–18)	Ongoing seizures 80%, death 20%
Singh et al. (81)	100%	8	120	None	2 and 3	12 and 18	Ongoing seizures, cognitive deficits
Thakur et al. (28)	90%	3	Median 16 (IQR 13–23, range 4–41)	Hypertriglyceridemia 20%, acidosis 10%	Median 4 (IQR 3–4, range 2–6)	–	Seizure freedom 10%, ongoing seizures 40%, death 20%
Caraballo et al. (82)	50%	–	–	–	–	–	Ongoing seizures
Sort et al. (83)	66.6%	1 and 13	Median 21 (range 15–28)	Weight loss 33.3%, hypertriglyceridemia 33.3%	Median 3	Median 6 (range 1–14)	Death 33.3%, ongoing seizures 33.3%, lost to FU 33.3%
Strzelczyk et al. (70)	100%	4	–	None	–		Ongoing seizures
Martikainen et al. (71)	100%	5	60	None	1	2	Seizure freedom
Vaccarezza et al. (29)	80%	Median 2 (range 1–3)	Median 365 (range 10–720)	Diarrhea 40%, hypokalemia 20%	–	Median 12.1 (range 0.3–24)	Death 20%, ongoing seizures 60%, lost to FU 20%
Cervenka et al. (72)	100%	11	90	None	3	3	Seizure freedom
Ismail and Kossoff (2011)	100%	10	150	None	3	5	Ongoing seizures
Kramer et al. (7)	14.3%	2	–	–	–	–	Death 14.3%, cognitive deficits 85.7%
Nam et al. (30)	100%	Median 8 (IQR 7–14, range 3–19)	Median 150 (IQR 30–240, range 30–480)	Hypertriglyceridemia 20%, constipation 80%, gastroesophageal reflux 40%, aspiration pneumonia 20%	–	Median 5 (IQR 3–8, range 1–16)	Ongoing seizures 40%
Kumada et al. (84)	100%	5 and 10	570 and 120	None	–	18 and 4	Ongoing seizures 50%, seizure freedom 50%
Nabbout et al. (31)	77.8%	Median 5 (IQR 4–6, range 4–6)	540	–	–	18	Death 11.1%, ongoing seizures 88.9%
Wusthoff et al. (85)	100%	6 and 4	365	None	3 and 4	12	Seizure freedom 50%, lost to FU 50%
Villeneuve et al. (11)	80%	Median 2.5 (IQR 1.5–6.5, range 1–10)	Median 180 (IQR 30–130, range 21–360)	Severe vomiting 80%, asthenia 60%, severe anorexia 20%, non-symptomatic hypoglycemia 80%, drowsiness 60%	–	Median 6 (IQR 2–12, range 1–20)	–
Bodenant et al. (74)	100%	6	77	None	4	2.5	Death
Baumeister et al. (75)	0%	–	3	Fatal propofol infusion syndrome	6	–	Death
François et al. (32)	50%	–	Median 79 (range 9–98)	Weight gain 33.3%, height-weight stagnation 66.6%, digestive disorder 66.6%, hypoglycemia 33.3%, renal lithiasis 16.6%, asthenia 83.3%, sinus dysfunction of central origin 16.6%, cardiac arrest (hypokalemia) 16.6%	–	Median 0.3 (range 0.3–2)	Seizure freedom 50%

ASMs, antiseizure medications; DD, developmental delay; DR, drug resistant; FU, follow-up; ID, intellectual disability; IQR, interquartile range; KD, ketogenic diet; mRS, Modified Ranking scale; No, number; SE, status epilepticus.

#### 3.2.1. Individual data extraction and analysis of the literature

Overall, the included studies described 276 patients, both of pediatric and adult age, receiving KD during SRSE. One hundred twenty-three/245 (50.2%) were female (information detailed in 71 studies). The majority of reported patients were children (208/276; 75.3%). The median age at SE onset was 9.1 years old [interquartile range (IQR)]: 5.2–20 years; range: 1.2 months−73 years; information available in 73 studies].

#### 3.2.2. Etiology details

The most common etiology of SRSE was acute symptomatic (59/276; 21.3%), among these patients, 67.7% (40/59) presented with NORSE of immune (25/40; 62.5%) and infectious (15/40; 37.5%) etiologies. Other etiologies were remote symptomatic (19/276; 6.8%), progressive symptomatic (17/276; 6.1%), and SE in defined electroclinical syndromes (41/276; 14.8%), including two patients with genetic etiology and NORSE presentation. The etiology was unknown in 50.7% of the patients (140/276) presenting with NORSE, of which 102 presented with FIRES.

#### 3.2.3. Treatment details and response to ketogenic diet

Overall, the median time duration of SRSE before KD initiation was 9 days (IQR: 5.2–20; range: 1–73; information available in 56 studies), the median number of treatments (ASMs and anesthetics) prior to KD was 6 (IQR: 5–8; range: 2–14; information detailed in 69 studies), and 143/276 (51.8%) patients also received other treatments prior KD mostly including immunotherapy (133/143; 93%).

KD was considered effective in 197/276 (71.4%) patients after a median time from KD initiation of 6.5 days (IQR 4–9, range 1–28). Overall, the total length of KD was 60 days (IQR 21–180; range: 3–900) in responders and non-responders patients (information available in 47 studies).

In patients with NORSE presentation (182/276, 65.9%), the median time of duration of SRSE before KD initiation was 15 days [interquartile range (IQR): 9–28; range: 2–420], and the median number of other treatments prior to KD was 7 (IQR: 5–8; range: 2–16). KD was considered effective in 117/182 (64.3%) after a median time from KD initiation of 8 days (IQR 6–21, range 1–30).

Overall, adverse effects due to KD were reported in 124/276 (44.9%) patients.

#### 3.2.4. Outcomes

Twenty-seven out of 276 (9.7%) patients died during the acute phase of SRSE, while 7/276 (2.5%) died after achieving SE cessation. At the latest follow-up with a median length after SE cessation of 10 months (IQR 3.3–18, range 9 days−156 months), 50/242 (20.6%) patients achieved seizure freedom, 46/242 (19%) suffering from ongoing seizures, while 44/242 (18.2%) had ongoing seizures associated with cognitive impairment, and 33/242 (13.6%) had cognitive impairment alone (information available in 63 studies). Overall, 88/90 (97.7%) with ongoing seizures received a median number of ASMs of 3 (IQR: 3–4, range: 1–10; information available in 36 studies).

### 3.3. Ketogenic diet effectiveness and influencing factors

For this analysis, we considered the literature cases in addition to our center cases. The data of 255/292 (82.9%) patients were available for Kaplan–Meier survival curves.

The probability to achieved SRSE cessation after KD initiation is 50.53% at 7 days [95% confidence interval (CI): 44.15–56.57], 33.16% at 14 days (95% CI: 27.21–39.22), and further decreases to 26.34% at 21 days (95% CI: 20.77–32.22), and 25.24% at 28 days (95% CI: 19.73–31.10).

The KD responder rates are different in children compared to adults (HR: 1.47, 95% CI: 1.04–2.06; *p* < 0.02). The median time to achieve SRSE cessation after starting KD is 8 days in children (IQR: 6–16; range: 1–30) and 5.5 days in adults (IQR 3–10; range 1–30).

A previous history of epilepsy implies a greater likelihood of KD efficacy in achieving SRSE cessation (HR: 1.54, 95% CI: 1.11–2.12; *p* = 0.009). The detection of known etiology implies a favorable response to KD (HR: 1.70, 95% CI: 1.26–2.30; *p* < 0.0001); in this regard, patients with an acute symptomatic cause of SRSE have a greater likelihood of KD efficacy (HR: 1.58, 95% CI: 1.13–2.23; *p* = 0.008).

**Figure 2:**
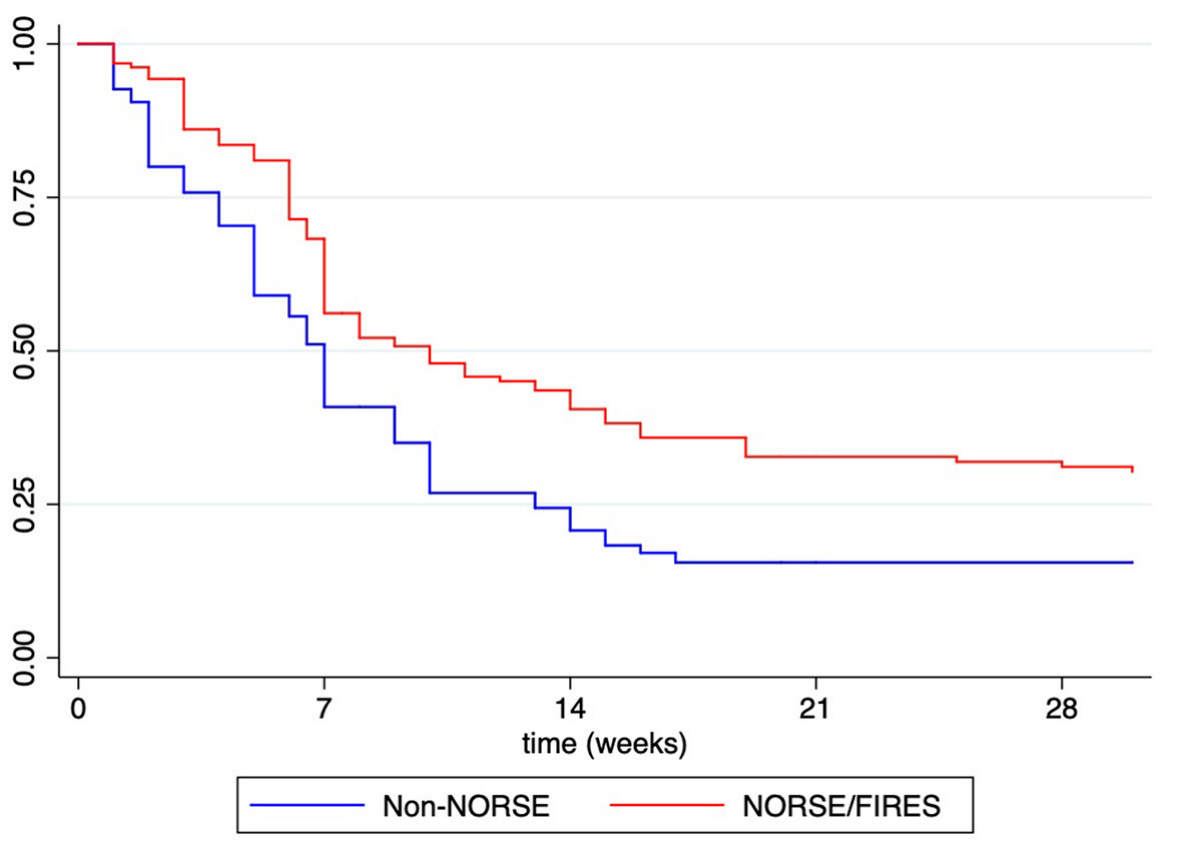
Kaplan–Meier survival estimates for probability to achieved RSE cessation after KD initiation in patients with NORSE/FIRES presentation vs. non-NORSE (other SRSE).

**Figure 3:**
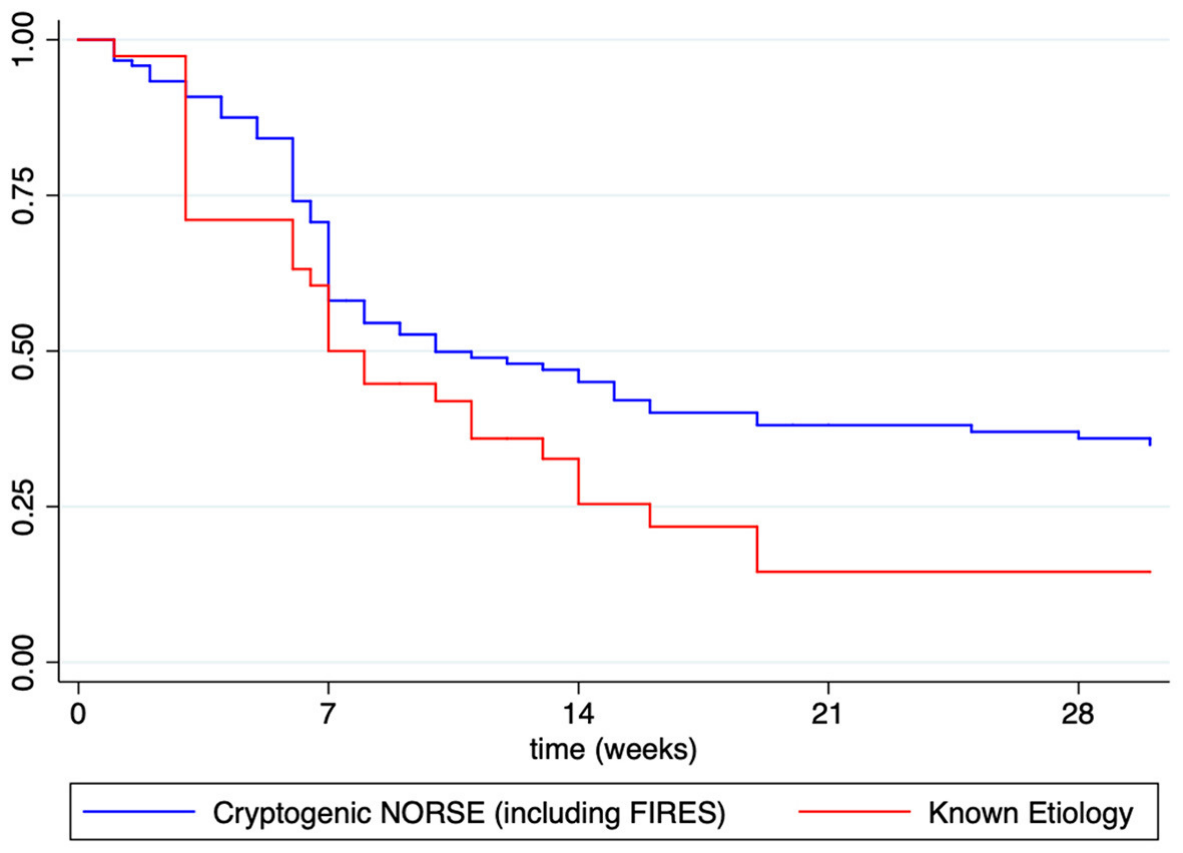
Kaplan–Meier survival estimates for probability to achieved NORSE cessation after KD initiation in patients with known etiology vs. cryptogenic NORSE (including FIRES).

Otherwise, even though most patients with NORSE benefit from KD (117/185, 63.2% achieving SRSE cessation, *p* < 0.004), they needed, however, a longer time to achieve SE resolution after starting KD compared with other non-NORSE SRSE (HR: 0.60, 95% CI: 0.44–0.81; *p* = 0.001; Figure 2). At the Kaplan–Meier survival analysis, the probability of achieving NORSE cessation after KD initiation was 56.13% at 7 days (95% CI: 48.02–63.48), 40.51% at 14 days (95% CI: 32.58–48.28), 32.75% at 21 days (95% CI: 25.20–40.50), and 31.12% at 28 days (95%CI: 23.66–38.85). The response to KD in the NORSE group with identified etiology compared to the cryptogenic NORSE was significantly higher (*p* = 0.01), and the time to achieve SE resolution after starting KD was shorter (HR: 1.56, 95% CI: 1.01–2.38; *p* = 0.04; Figure 3).

Overall, the number of treatments before KD initiation has a negative impact on the responder rate (HR: 0.93, 95% CI: 0.88–0.98, *p* = 0.01), while the time from SRSE onset to KD initiation does not significantly impact KD efficacy (HR: 0.99, 95% CI: 0.99–1.00, *p* = 0.23).

Side effects of KD negatively impact the probability to achieve SRSE cessation after KD initiation (HR: 0.60, 95% CI: 0.44–0.81, *p* = 0.001). Other KD related factors such as fasting before diet initiation, KD ratio, time to reach ketosis, and total length of KD, do not impact the likelihood of KD efficacy in achieving SRSE cessation.

## 4. Discussion

SRSE is a major neurological emergency, and the therapeutic interventions aim to reduce its duration, mortality, as well as short- and long-term comorbidities. NORSE (with its subcategory FIRES) is one of the most common causes of SRSE. Therapeutic alternatives are scarce, and the use of anesthetic agents as symptomatic treatment could worsen the outcome due to systemic complications that often co-occur. So far, there is currently no high-level evidence to guide NORSE management since most of the therapeutic approaches come from expert opinions and few cases. A few studies and case series on immunotherapy with monoclonal antibodies efficacy have been reported so far (40, 43, 47, 53, 59, 87), but their effectiveness has still to be assessed in large cohort studies.

NORSE outcomes are influenced mainly by non-modifiable variables such as age and underlying etiology, though complications from the NORSE status itself, treatments, and length of stay in ICU also contribute to morbidity and mortality.

KD is an emerging treatment option for RSE and SRSE (88), and most published evidence has shown high efficacy rates (12, 13, 89). The multiple mechanisms of action make KD a good therapeutic option in these conditions. The anti-seizure effect of KD may be due to multiple mechanisms involving neurotransmitters, mitochondria, gut microbiota, DNA methylation, ion channels, inflammation, and G-protein coupled receptors. It mimics ASMs polytherapy (88), and several of these mechanisms can occur rapidly, while others, such as the effects on mitochondria, gut microbiota, and DNA methylation, are likely long-term.

Many case reports and case series have demonstrated the potential efficacy and safety of KD for the acute treatment of SRSE; however, the quality of these studies remains scarce.

More literature reports the use of KD in children compared to adults, but studies on the adult population have shown higher efficacy rates (87.5 vs. 66.8%; *p* = 0.001) and a shorter time to achieve SE cessation after starting KD. This discrepancy is probably due to more refractory cases being over-represented in the childhood population.

In this systematic review, about half of patients experiencing SRSE, a cause has been identified, and almost a quarter have a previous history of epilepsy. However, half of the cases elude any easily detectable etiology, and previously healthy individuals develop prolonged NORSE without a readily identifiable explanation. Overall, patients with SRSE of known etiology appear to present a better response rate and a shorter time to achieve SRSE cessation after starting KD. SE occurring during the course of epilepsy syndromes, such as genetic and structural epilepsies, may benefit from KD in 75% of the cases. Furthermore, patients with SRSE of remote etiology were also reported as responders to KD in 62.5% (11, 26, 64, 74). Patients with SRSE due to progressive etiologies such as mitochondrial diseases (71) are good candidates for KD to be introduced early. Other etiologies involving immune-mediated pathways, such as Rasmussen encephalitis and autoimmune encephalitis with SRSE were reported to benefit of KD (18, 19, 28, 56, 57, 61, 66, 80, 85). In this regard, the presumed immune etiology in FIRES and NORSE cases, based on the activation of an inflammatory cascade, makes these conditions possible specific targets for KD (90). In this systematic review, NORSE, and its subcategory FIRES, are common causes of SRSE, but these difficult-to-treat conditions imply a longer time to achieve SE resolution after starting KD compared to other SRSE. This might be due to the addition of specific treatment tailored for etiologies and the high level of cases remaining without an etiology (cryptogenic NORSE) or where etiology was much delayed.

The etiology remains unexplained in about two-thirds of the cases of NORSE, representing the so-called “cryptogenic NORSE.” The most identified cause in adult patients is autoimmune encephalitis, while infections are the prevalent etiology in pediatric patients (91).

The analysis of literature data combined with our single center experience highlighted a more favorable response to KD and a shorter SE duration in the NORSE group with identified etiology compared with NORSE of unknown etiology. These findings highlight the alternative therapeutic role of KD in patients affected by NORSE and FIRES, even though this favorable response needs to be better evaluated and confirmed in prospective controlled studies assessing both seizure control and functional outcome. The detection of an underlying cause may also allow an early treatment at the pathogenic level, which may reduce the risk of irreversible sequelae in the long-term. The recent international consensus recommendations for the management of NORSE, including FIRES, provides diagnostic and therapeutic algorithms to aid clinicians in patient care (92, 93). The consensus recommends the initiation of the KD in the first week, or if not already given, KD should be considered in prolonged and severe cases, emphasizing the importance of starting KD very early in the course of NORSE. These management recommendations may allow a faster and more tailored diagnostic process and improve treatment to allow better outcomes.

The main limiting factor for the use of KD in NORSE might be the time lag for efficacy, ketosis is usually reached within 24–72 h, and seizure reduction within the first week in the majority of the patients. This time lag could be challenging to accept in a severe condition such as NORSE.

Another impeding factor for the initiation of KD highlighted by several panelists of the consensus (92) is the limited availability and the lack of experience in its administration, particularly in adult patients. However, the expertise on KD in adult neurology is still increasing, and the number of adult patients with epilepsies, mostly of genetic etiology, treated with KD is on the rise.

The KD is well-tolerated with low rates of side effects in the ICU setting, highlighting that the diet has a safe profile and should be implemented in these settings. The most frequently reported side effects are easily manageable gastrointestinal or biochemical abnormalities, and the few serious adverse events reported in the literature are not necessarily attributable to KD.

The feasibility of implementing the KD in ICUs may be challenging also due to intensive care procedures, the possible occurrence of severe adverse events, and the concurrent administration of glucose-containing medications. A multidisciplinary team, including experienced physicians and dietitians, and standardized protocols should be warranted in these settings to overcome these issues. Most survivors have long-term sequelae in terms of drug-resistant epilepsy and poor functional outcomes, mostly related to the length of stay in the ICU and underlying etiology.

Due to its emergent and rare nature and the heterogeneity of the causes, randomized controlled treatment trials in NORSE are scanty. Literature data on KD in SRSE and NORSE comes mainly from retrospective observational studies, small case series, and anecdotal case reports that mainly report the good efficacy of the diet and rarely detail its failure. These studies have inherent limitations and heterogeneity in etiology, protocols, and assessment criteria. Treating NORSE involves multiple medications and treatments given together, making it difficult to impute SRSE termination to a single therapeutic agent directly. In this regard, it is difficult to assess the primary therapeutic effect of KD or its synergistic action with other treatments. Furthermore, in some patients receiving concurrent medications targeting an underlying etiology, the resolution of SRSE cannot be directly attributed to the KD only. In this regard, the evidence of these reports shares the same weakness with all third-line treatments in RSE and SRSE, where no agent has achieved a high level of evidence-based medicine (3).

Although promising, the current results should be interpreted with caution due to the inherent bias, confounding factors, and small sample size of the included studies.

Evidence-based medicine is dramatically lacking to date, particularly in critical situations such as ICUs. In this regard, prospective, randomized controlled trials are needed to better assess KD as third-line therapy in managing RSE and preventing SRSE, mostly in patients with NORSE presentation. They should evaluate KD effectiveness in these specific settings, identify predictors of treatment response, and determine a ratio-responsive relationship of treatment. Outcomes should be assessed in the short-term, considering SE resolution, and in the long-term, evaluating subsequent seizure burden and neurological functioning.

## Data availability statement

The raw data supporting the conclusions of this article will be made available by the authors, without undue reservation.

## Ethics statement

Ethical review and approval was not required for the study on human participants in accordance with the local legislation and institutional requirements. Written informed consent to participate in this study was provided by the participants' legal guardian/next of kin.

## Author contributions

RN and SM contributed to the study concept, data acquisition and analysis, and drafting of the manuscript. PD contributed to the data acquisition and drafting of the manuscript. OD and MO contributed to the study concept, data acquisition, and drafting of the manuscript. All authors contributed to the article and approved the submitted version.
